# A One Health Message about Bats Increases Intentions to Follow Public Health Guidance on Bat Rabies

**DOI:** 10.1371/journal.pone.0156205

**Published:** 2016-05-25

**Authors:** Hang Lu, Katherine A. McComas, Danielle E. Buttke, Sungjong Roh, Margaret A. Wild

**Affiliations:** 1 Department of Communication, Cornell University, Ithaca, New York, United States of America; 2 Wildlife Health Branch, Biological Resources Division, National Park Service, Fort Collins, Colorado, United States of America; 3 Lee Kong Chian School of Business, Singapore Management University, Singapore, Singapore; Institute for Health & the Environment, UNITED STATES

## Abstract

Since 1960, bat rabies variants have become the greatest source of human rabies deaths in the United States. Improving rabies awareness and preventing human exposure to rabid bats remains a national public health priority today. Concurrently, conservation of bats and the ecosystem benefits they provide is of increasing importance due to declining populations of many bat species. This study used a visitor-intercept experiment (N = 521) in two U.S. national parks where human and bat interactions occur on an occasional basis to examine the relative persuasiveness of four messages differing in the provision of benefit and uncertainty information on intentions to adopt a rabies exposure prevention behavior. We found that acknowledging benefits of bats in a risk message led to greater intentions to adopt the recommended rabies exposure prevention behavior without unnecessarily stigmatizing bats. These results signify the importance of communicating benefits of bats in bat rabies prevention messages to benefit both human and wildlife health.

## Introduction

With the highest case fatality rate of any infectious disease of humans, rabies remains an important public health concern in the United States [1, 2]. Reduction in rabies in domestic cats and dogs thanks to successful rabies vaccination campaigns since 1980 has meant that, today, more than 90 percent of reported rabid animals in the United States are wildlife [3]. Further, most human deaths caused by rabies in the past several decades have been associated with exposures to rabid bats [4].

Since vaccination of bats against rabies is not currently feasible, secondary intervention methods, such as public health education and exposure prevention, are the current means of preventing human infection with bat rabies [3]. Accordingly, improving rabies awareness among health care providers and the general public remains a high priority [1, 5] in that educational messages about how to prevent, recognize, and treat potential contact with rabid bats are crucial in reducing human deaths [1].

Concurrently, the conservation of bats and the ecosystem benefits they provide is of increasing importance due to declining populations of many species [6]. It is therefore imperative that wildlife-associated disease messages use an evidence-based approach to ensure that they do not produce unintended consequences that might diminish public support for wildlife [7]. Risk messages often center on negative information that is inherently fear arousing [8]. While this strategy is commonly used in public health messaging, its effectiveness for public health outcomes associated with wildlife disease is less known, and some research suggests it might even have negative impacts on wildlife conservation [7].

An approach that seeks to reformulate public or wildlife health initiatives under a larger framework is One Health. One Health focuses on the interdependence of human, domestic animal, wildlife, and ecosystem health and advocates an approach that enhances the health of all species [9]. In the context of disease messaging, a One Health approach would be exemplified by messages that convey both negative (e.g., risks associated with bat rabies) and positive (e.g., ecological benefits of bats) information [10]. However, because a One Health approach to risk messaging has received little evaluation, further research is warranted to test messages that seek to motivate wildlife-associated disease prevention behaviors while at the same time sustain general support for wildlife [7, 10]. In particular, the current study seeks to examine a One Health approach to messaging about bats and rabies to examine the effects of such messages on important public and wildlife health outcomes.

In this regard, we first examine the relative persuasiveness of simultaneous communication of both risk and benefit information (i.e., the risk-benefit message), versus communication of mere risk information (i.e., the risk-only message), in relation to intentions to adopt a bat rabies exposure prevention behavior in a U.S. national park. Studies in the wider risk communication literature have found inconsistent results regarding the usefulness of risk-benefit messages [11]. On the one hand, risk-benefit messages may provide more balanced information for individuals to make informed decisions on a health risk issue [12]. On the other hand, messages incorporating both risks and benefits may seem incongruent to audiences and decrease the credibility of or distract audiences from the prevention messages [13]. These inconsistencies further warrant the investigation of risk-benefit message efficacy in communicating bat rabies public health guidance.

Second, we examine the influence of the level of uncertainty associated with the risk [14]. Uncertainty can relate to perceptions of the temporal distance of a risk. For example, the probability of exposure to a health risk may be perceived higher when similar risks are prominent more recently than in the distant past [15, 16]. Testing the impacts of different levels of risk uncertainty conveyed in a bat rabies-related message has important practical considerations. As risk uncertainty levels differ across real-world situations, it is necessary to examine if messages capturing these variations will lead to different outcomes. For instance, certain levels of uncertainty may be more likely to result in unnecessary fear and strong denial of the message [17].

We conducted a field experiment in two U.S. national parks. We assessed the impact of bat rabies risk messages on park visitors’ intentions to adopt appropriate rabies prevention behaviors. We manipulated messages in terms of the benefits of bats and the risk uncertainty associated with bat rabies. Our objectives were to determine if: 1) messages including the benefits of bats impacted public health prevention intent, and 2) risk uncertainty influenced public health prevention intent.

## Materials and Methods

Prior to the start of this study, the Office of Research Integrity and Assurance at Cornell University reviewed and approved this research including the consent procedure, as did the National Park Service, in consultation with the U.S. Department of Interior’s Office of Management and Budget.

Trained research assistants approached visitors in the park and asked for their participation in the study if they were older than 18. If a group was encountered, the assistants asked the individual within the group who had the next birthday to serve as the respondent. Before starting the study, participants were informed that completion of the survey implied their consent to participate in this study. Therefore, the completion of the survey was regarded as written consent. We did not ask for participants’ names to ensure anonymity and avoid any identifiable association between participants and their responses.

We recruited a total of 521 visitor participants from two national parks (258 from Mammoth Cave National Park in Kentucky and 263 from Great Smoky Mountains National Park in Tennessee) in late July 2014. These parks were selected based on two criteria. First, visitors in these parks could get in close proximity with bat populations. Second, these parks have had previous bat-related public health issues, such as bat rabies and visitor-bat contact.

### Design

Each participant was randomly assigned to either a control (no message) condition or one of four message conditions as part of a 2 (risk-benefit vs. risk-only) x 2 (low uncertainty vs. high uncertainty) between-subjects factorial design. In the message conditions, participants read one of four printed leaflets at the beginning of the experiment. The leaflets, which were designed to resemble flyers usually seen in a park and checked for accuracy by wildlife and public health professionals, mentioned the park’s history of rabid bats, described the negative consequences of exposure to bat rabies, and recommended telling a park ranger right away should visitors encounter a bat that is behaving strangely. To distinguish between risk-benefit and risk-only messages, the risk-benefit messages included an additional description of the ecological benefits of bats and the One Health tenet, “Our health is linked to the health of all species and the environment.” We manipulated the high versus low risk uncertainty by varying the temporal distance of a rabid bat incident. The high uncertainty messages stated, “This park has had rabid bats in the past,” whereas the low uncertainty messages stated, “A rabid bat was recently found in this area of the park.” After participants finished reading the leaflets, they received a printed questionnaire to complete. Those in the control condition completed the questionnaire without having read any leaflets.

### Measures

The primary outcome variable of interest was the likelihood of participants telling a park ranger about a strangely behaving bat should they see one (where 1 = “very unlikely” and 5 = “very likely”). This behavior, which benefits not only the individual adopting this behavior but also other park visitors, was promoted as the bat rabies exposure prevention behavior in the leaflets.

In addition, we measured participants’ beliefs about bats using six items. Participants were asked to indicate the extent to which they agreed or disagreed that bats were attractive, vulnerable, worthless (reverse-coded), harmless, native and frightening (reverse-coded) on a 5-point scale (where 1 = “strongly disagree” and 5 = “strongly agree”). Responses to these six items were averaged to create a belief composite scale.

For participants in the message conditions, we measured their evaluation of the messages using three items. Participants were asked to indicate the extent to which they agreed or disagreed that the message they read was clear, informative, or persuasive on a 5-point scale (where 1 = “strongly disagree” and 5 = “strongly agree”). These items were included to explore the underlying mechanism for potential message effects.

### Analysis

We first examined if the experimental randomization was successful by testing for differences in participants’ characteristics across conditions with the *χ*^*2*^ test and analysis of variance (ANOVA). Next, we used a two-way ANOVA with benefit frame (risk-benefit vs. risk-only), uncertainty frame (high vs. low), and their interaction as independent variables, and the likelihood of telling a park ranger about a strangely behaving bat and beliefs about bats as dependent variables to examine the main and interaction effects of message frames. In addition, to compare each message condition with the control condition, we regressed the behavioral intention and belief variables respectively by using ordinary least squares regression on the dummy variables for the five conditions (control condition as the reference group). We also tested whether message effects on the behavioral intention were mediated respectively by perceived clarity, persuasiveness, or informativeness of the messages. We ran mediation analysis using the PROCESS macro (5000 bootstrap resamples, 95% bias corrected CI) [18].

## Results

The sample was 50.1% female, 89.8% White, 4.2% Asian, 1.7% African American, with a mean age of 44.78 years (*SD* = 14.34). Forty-five percent of the sample had completed less than a college education, and 14.0% had completed only high school or less. Seventeen percent of the sample reported annual household incomes under $35,000. Eighty-one percent of the sample had visited a national park site before this particular visit. Three percent of the sample reported having seen a rabid bat, and eight percent reported being unaware that some bats carry rabies. The total sample in each condition was as follows: risk-benefit/low uncertainty (*n* = 113), risk-benefit/high uncertainty (*n* = 109), risk-only/low uncertainty (*n* = 99), risk-only/high uncertainty (*n* = 103), and control (*n* = 97).

First, we confirmed that the experimental randomization was successful by finding no significant differences across conditions in participants’ age, gender, race, education, annual household income, experience with rabid bats, awareness of bat rabies, and visits to national park sites (Table 1).

**Table 1:** Demographic characteristics of participants in a visitor-intercept experiment to assess responses to messages about bat rabies exposure prevention.

	Test of randomization across 5 groups
Age (mean)	F(4, 508) = .67; *p* = .62
Female	Pearson *χ* ^2^(4) = 4.61; *p* = .33
White race	Pearson *χ* ^2^(4) = 4.62; *p* = .33
Education < Bachelor’s degree	Pearson *χ* ^2^(4) = 1.20; *p* = .88
Household income <75, 000	Pearson *χ* ^2^(4) = .56; *p* = .97
Having seen a rabid bat	Pearson *χ* ^2^(4) = 2.16; *p* = .71
Not aware that some bats carry rabies	Pearson *χ* ^2^(4) = 5.68; *p* = .22
Having visited a national park site before	Pearson *χ* ^2^(4) = 3.01; *p* = .56

The results of our two-way ANOVAs indicate a significant main effect of the benefit frame on participants’ likelihood of telling a park ranger should they encounter a strangely behaving bat (*M* = 4.31, *SD* = .97), *F*(1, 419) = 4.62, *p* < .05, *η*^2^ = .01. Particularly, participants who read the risk-benefit messages (*M* = 4.41, *SD* = .90) showed stronger intentions to adopt the recommended behavior than those who read the risk-only messages (*M* = 4.21, *SD* = 1.04). We found no other significant main or interaction effects for this dependent variable or the beliefs about bats scale (Cronbach *α* = .72; *M* = 3.61, *SD* = .62).

Comparing each of our message conditions with the control condition, we found that the risk-benefit/low uncertainty and the risk-benefit/high uncertainty messages increased intentions to tell a park ranger about a strangely behaving bat; further, none of the messages elicited significantly more negative beliefs about bats (Table 2).

**Table 2:** Message effects on intention to tell a park ranger about a strangely behaving bat and beliefs about bats.

Message	Behavioral Intention, B(SE)	Beliefs about Bats, B(SE)
Risk-benefit/low uncertainty (Ref = control)	.28* (.14)	-.12 (.09)
Risk-benefit/high uncertainty (Ref = control)	.34* (.14)	-.08 (.09)
Risk-only/low uncertainty (Ref = control)	.18 (.14)	-.05 (.09)
Risk-only/high uncertainty (Ref = control)	.03 (.14)	-.17 (.09)
Intercept	4.10*** (.10)	3.70*** (.06)

*, **, *** Significant at the *p* = .05, *p* = .01, and *p* = .001 level, respectively.

**Fig 1:**
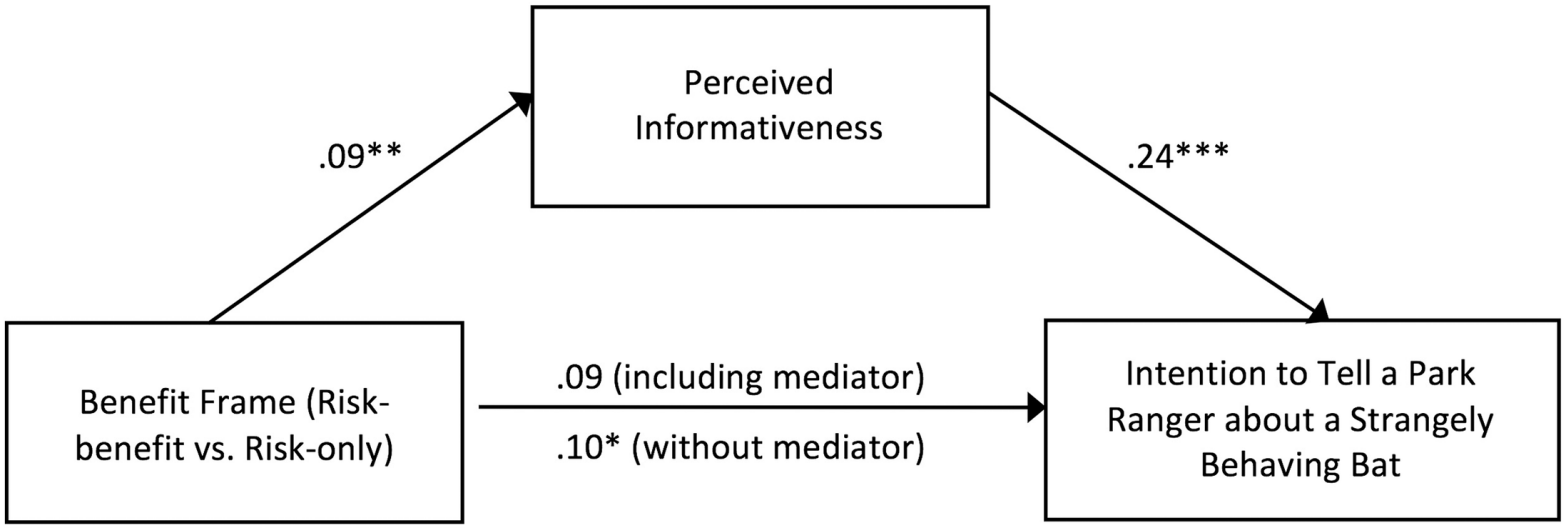
Mediation analysis investigating the relationship between benefit frame, perceived informativeness, and intention to tell a park ranger about a strangely behaving bat. Note: Total indirect effect = .02 (95% confidence interval = .01, .05). The uncertainty frame and the interaction term between the benefit frame and the uncertainty frame are not shown to reduce visual clutter. *p < .05; **p < .01; ***p < .001.

We also examined the underlying mechanism accounting for the main effect of the benefit frame. Our mediation analyses found that the perceived informativeness (*M* = 4.39, *SD* = .68) rather than perceived persuasiveness (*M* = 3.91, *SD* = .88) or clarity (*M* = 4.41, *SD* = .63) of the messages fully mediated the relationship between benefit frame and intentions to tell a park ranger about a strangely behaving bat when controlling for uncertainty frame and their interaction term (Fig 1). More specifically, adding benefit information to the risk-only messages increased the perceived informativeness of the messages and thus further increased the likelihood of participants adopting the recommended behavior in the messages.

## Discussion

Encroachment of human development on wildlife habitat and habitat loss have led to increased interactions between wildlife and humans. However, close-range interactions can sometimes have negative consequences for both wildlife and human health [19]. As bat rabies variants have become the greatest source of human rabies deaths in the United States since 1960, public health communication efforts focused on preventing human contact with rabid bats comprise a crucial component of effective rabies prevention and control programs [1]. This is true in general settings as well as specific ones, such as U.S. national parks.

Our results of a field experiment examining bat rabies risk messaging in two national parks showed that messages including both risks and benefits of bats were more effective in promoting the specific public health recommendation than messages mentioning only risks from bats. Our comparison between message conditions and the control (no message) condition further confirmed that while the risk-only messages were not superior to the control condition, the risk-benefit messages *increased* participants’ intention to inform a park ranger of a strangely behaving bat. In addition, none of the four messages negatively influenced beliefs about bats relative to the control condition. Taken together, these findings suggest that the risk-benefit messages were more effective messaging strategies. These messages also reflect the One Health approach, which seeks to promote prevention behaviors to avoid wildlife-associated diseases while also maintain tolerance and support for wildlife [7, 10].

Why were risk-benefit messages more persuasive than risk-only messages? Our mediation analysis indicates that it was because participants perceived the risk-benefit messages as more informative. We believe that this perceived informativeness was not the by-product of any additional information in the message because the risk-only message conditions were no more efficacious than the control, no message condition. On the contrary, this perceived informativeness appears related specifically to the concept of One Health and the ecological benefits of bats provided in the risk-benefit messages. It is possible that because messages informed participants of contributions bats make to our environment, participants considered it more worthwhile to contact the park ranger should they encounter a strangely behaving bat. That is, such action would not only protect humans against rabies but also lower the chance of bats getting rabies or other infectious diseases from their own species.

We found no differential effects between the high versus low risk uncertainty messages on intentions to prevent bat rabies exposure. This finding has important practical implications. When warning visitors about potential bat rabies risks in the park, messages often mention the park’s history with such diseases. Our manipulation of uncertainty reflected the temporal distance or acuteness of the disease. The lack of difference between uncertainty frames indicates that these messages can be used in parks with recent rabid bat cases as well as those with a remote history of rabid bats. Put simply, general risk messages may be just as effective as specific risk messages with respect to when rabies was present in the park.

In viewing the results, we should consider study limitations. First, this experiment used a convenience sample of visitors in two national parks; therefore, the results should not be generalized to a broader public. However, we opted to test our messages in parks because large populations of both bats and people are found in many of the national parks, increasing the chance that people will encounter bats during their visits [5]. Additionally, it should also be noted that the primary goal of this study was to assess causal inference rather than representativeness. Future research seeking to confirm the external validity of our results should investigate similar messages with careful adaptions to other settings using a representative sample of the general population.

Second, we manipulated different levels of risk uncertainty by varying the temporal distance of past rabies incidence. While we could use other methods to accomplish similar manipulations (e.g., by varying the likelihood of a risk occurring), our chosen approach was relatively easy to incorporate and more accessible in our current messaging context; it has also been shown to influence perceived uncertainty in other contexts [15, 20]. It is important to note that the lack of a significant effect does not indicate that messages varying in risk uncertainty might have no effects in this context. Rather, it only suggests that the current manipulation had little impact. Future research can explore other ways of manipulating risk uncertainty in similar contexts to further examine its relevance.

Third, we used a single-item measure of behavioral intention, which may be less reliable than a multi-item measure. In addition, behavioral intention cannot be equated with actual behaviors, and thus the findings should be interpreted with extra caution when it comes to anticipating actual behaviors. Furthermore, we measured behavioral intention immediately after participants read the message, when the message effects might be strongest. Future research should explore the long-term (e.g., one hour or one day later) effects of these messages and their duration. Finally, we should point out that, while not uncommon in message testing studies, these findings should be interpreted with caution as they represent relatively small effects. Even so, it is noteworthy that relatively minor changes in the message wording showed effects, given the inability to control for all the competing messages visitors were exposed to in these parks.

Indeed, any risk communication effort in an uncontrolled setting, including national parks, can be quite challenging due to contextual factors introduced by the natural environment. Rather than abandon such messages to chance, we argue that these challenges further highlight the need for carefully designed messages that do not produce unintended effects. Public health risk messages understandably emphasize risks, but an overemphasis on risk can lead to unintended consequences that might conflict with and attenuate communication efforts [7]. This is especially the case when it comes to communication about wildlife-associated diseases, which by heightening awareness about risks could serve to decrease public support for wildlife. Our study suggests that a One Health approach that incorporates benefit information about bats alongside risk information may be more effective than risk-only messages. Therefore, incorporating risk-benefit messages into an overall approach may be a promising strategy for wildlife and public health professionals to communicate about wildlife-associated diseases, such as rabies.

## Supporting Information

S1 Dataset (SAV)
